# Supplementary material for: Ultrastrong nanocrystalline steel with exceptional thermal stability and radiation tolerance
Source: Nat Commun. 2018 Dec 19;9:5389. doi: 10.1038/s41467-018-07712-x (PMC6300597; doi:10.1038/s41467-018-07712-x)
Supplement: Supplementary file 1 — Supplementary Information [file 41467_2018_7712_MOESM1_ESM.pdf]

Supplementary Information

Ultrastrong nanocrystalline steel with exceptional thermal stability and radiation tolerance

Du et al.

Supplementary Figures

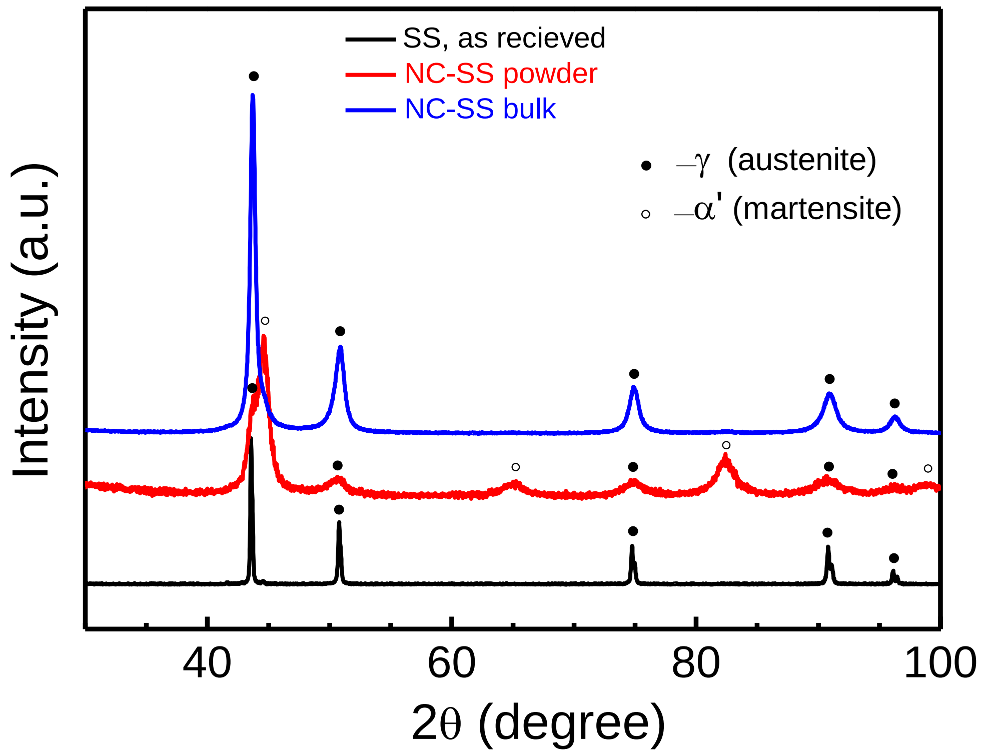

Supplementary Figure 1 | X-ray diffraction patterns of CG-SS (as received) and NC-SS powder and bulk. NC-SS powder was prepared by mechanically alloying CG-SS power and 1 at% La powder for 24 h. NC-SS bulk was prepared by consolidating the NC-SS powder under a pressure of 4 GPa and at a temperature of 1000 °C for 30 minutes.

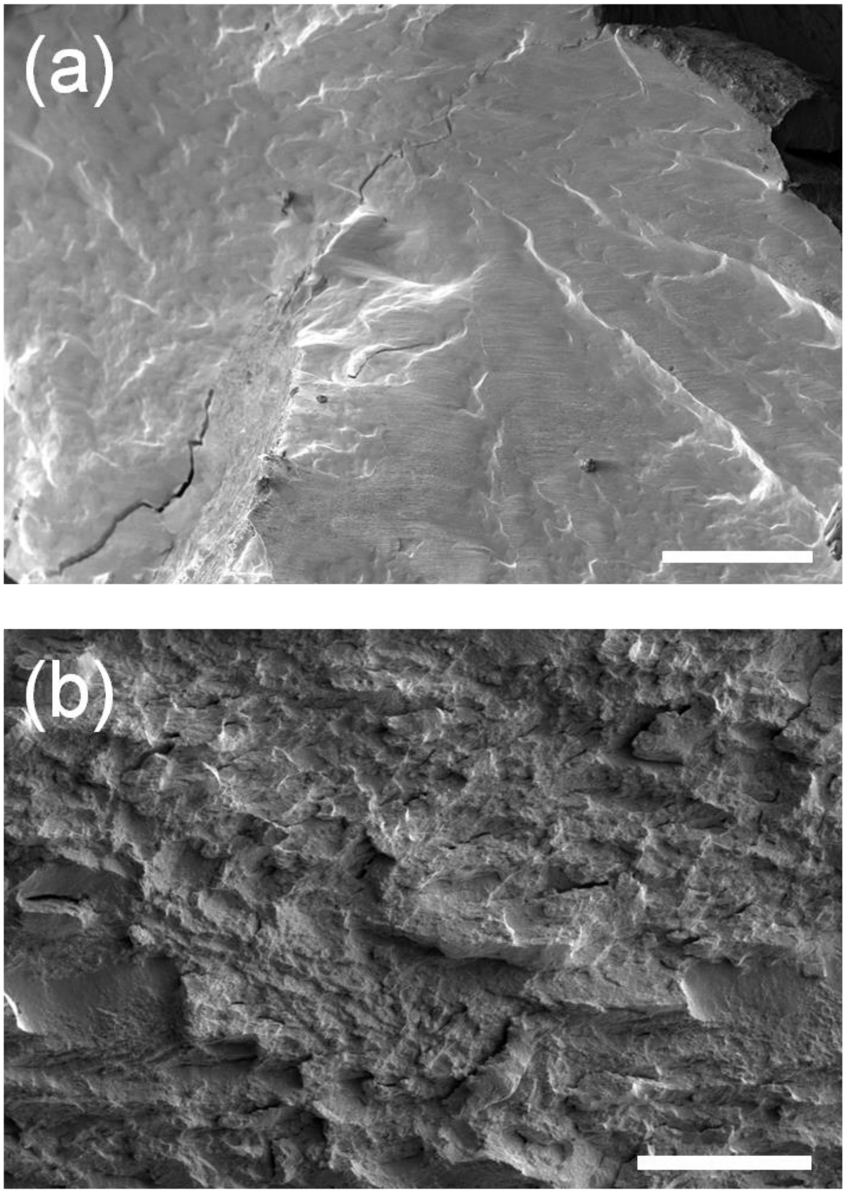

Supplementary Figure 2 | SEM images of NC-SS after compressive and tensile deformation. (**a**) Top surface of NC-SS cylinder after compressive deformation. Scale bar, 400 μm. (**b**) Fractured surface of NC-SS after tensile deformation. Scale bar, 25 μm.

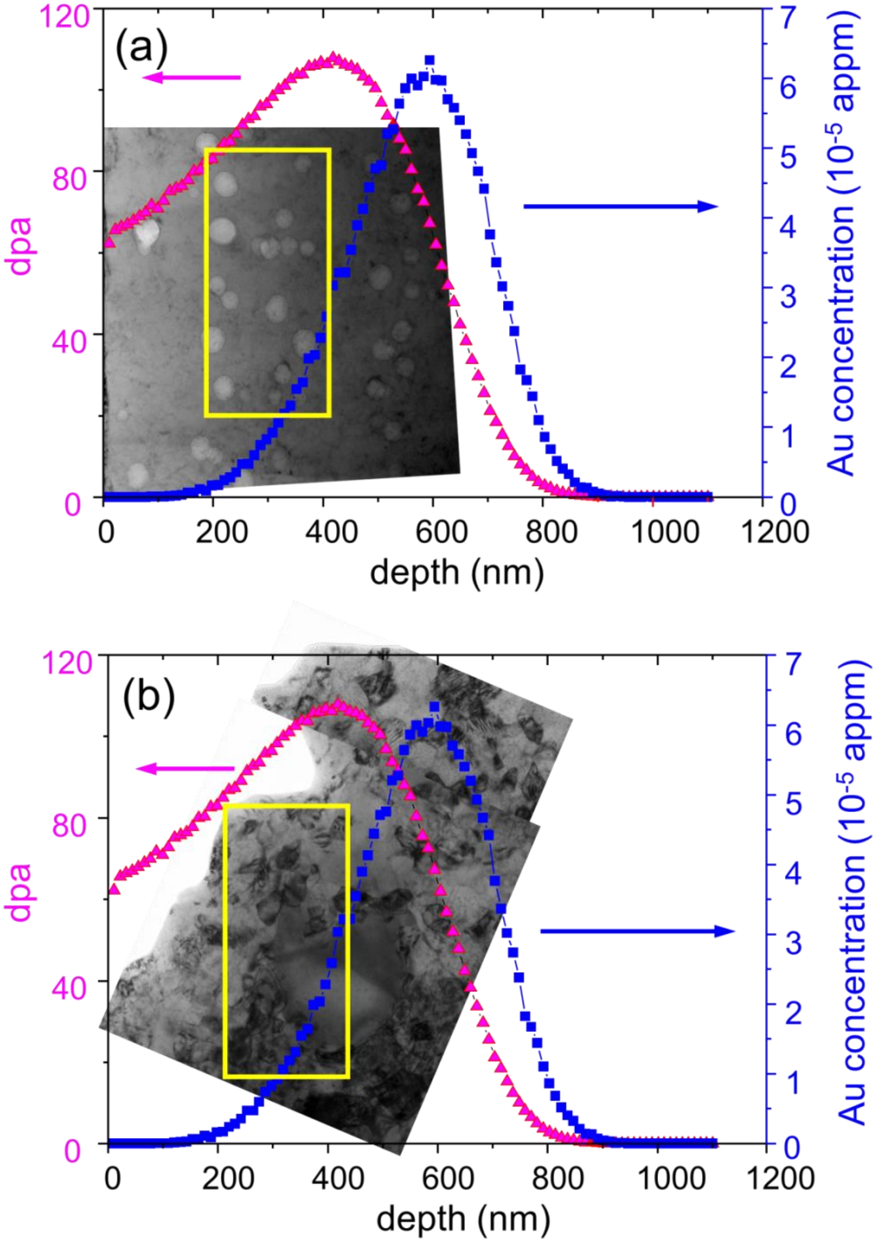

Supplementary Figure 3│TEM images and *ex situ* Au irradiation damage profiles. (**a**) CG-SS. (**b**) NC-SS. Fig. 1f and 1g are chosen in the area between ~ 200 and 400 nm away from the specimens' surface as indicated by the yellow boxes.

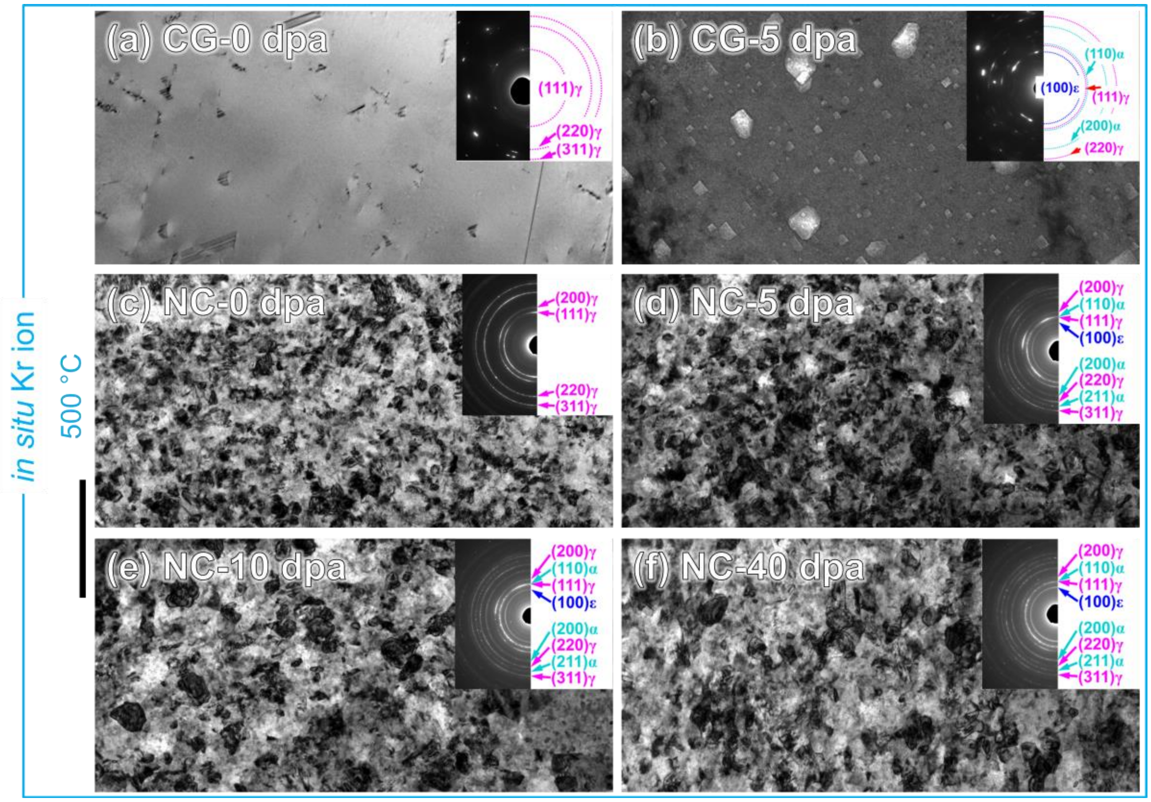

Supplementary Figure 4 | BF TEM images and the corresponding SAD patterns showing the overview of microstructure evolution of CG-SS and NC-SS after *in situ* Kr ion. (**a**) Before irradiation CG-SS has a single γ phase with giant grain size. (**b**) The formation of voids after *in situ* Kr ion irradiation of CG-SS to 5 dpa at 500 °C. New phases, bcc (α) and hcp (ε), appeared in the SAD patterns. (**c**) NC-SS also exhibits fcc phase before irradiation. (**d**) After 5 dpa of Kr ion irradiation, phase transformation in NC-SS also occurred. However, no voids were detected. After irradiation to10 dpa (**e**) and even 40 dpa (**f**), the microstructure remained nearly unchanged. Neither obvious grain growth nor void swelling was detected. Scale bar, 500 nm.

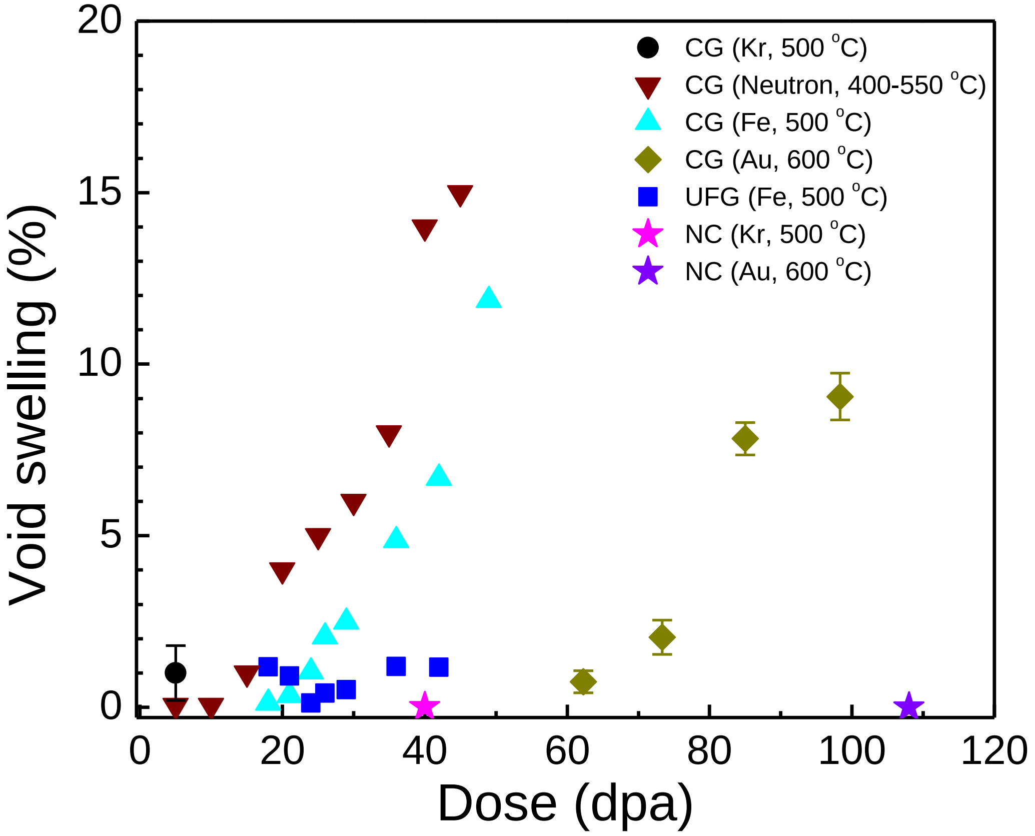

Supplementary Figure 5 | Comparison of void swelling of CG-SS, UFG-SS, and NC-SS irradiated between 400 and 600 ºC. The data for CG-SS are from Refs. 1, 2 and this study. The data for UFG-SS are from Ref. 1. The data for NC-SS are from this study. All SS are 304L type. Void swelling cannot be detected in NC-SS after both *in situ* Kr ion irradiation (40 dpa, 500 ºC) and *ex situ* Au ion irradiation (108 dpa, 600 ºC). In general, the void swelling in neutron irradiated bulk materials are derived from the measurement of dimensional changes, as the neutron radiation induced size change is often large enough (visible to naked eyes) to be measured in bulk specimens. For the heavy ion or Helium irradiated specimens, the magnitude of dimensional change is often very small. A profilometer is often used to measure the step height changes and then back calculate swelling[3,4]. Another widely used technique to measure small dimensional change is to use TEM[3,5]. In the current study, for both in situ and ex situ experiments, the TEM method is used to probe void swelling. The error bars are derived from calculating void size and density from multiple TEM micrographs. Approximately 100 voids are counted for each data point. Error bars, s.d..

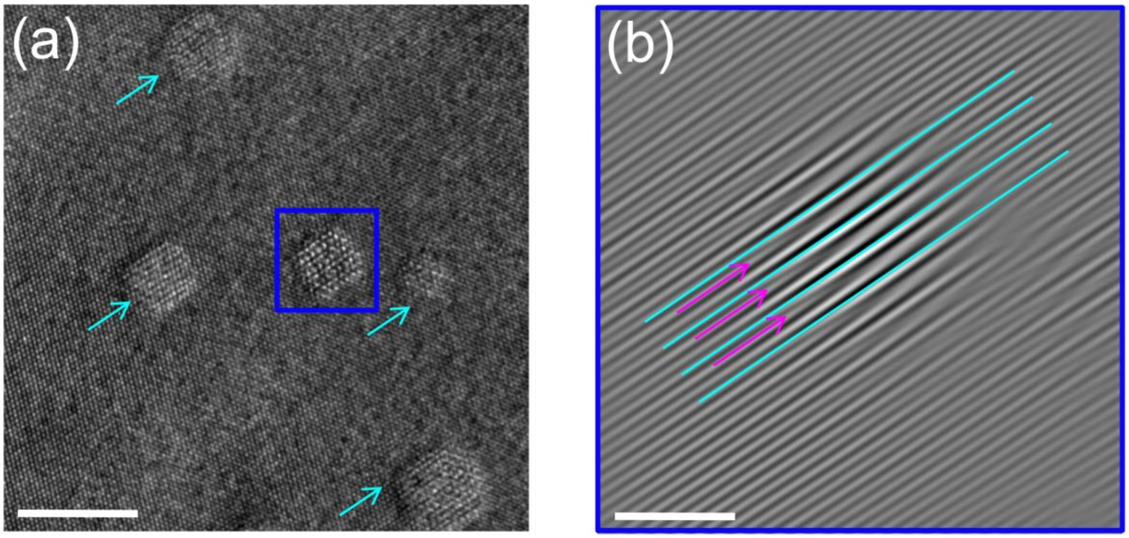

Supplementary Figure 6 | Microstructure of nanoprecipitates in NC-SS. (**a**) HRTEM image of NC-SS. Green arrows point out the precipitates in NC-SS. Scale bar, 5 nm. (**b**) Inverse fast Fourier transformation of the blue-boxed region in (**a**) highlighting that the precipitate and the matrix are semi-coherent. The magenta arrows and turquoise lines show the mismatch between the precipitate and the matrix, namely, every two atomic planes in the precipitate match one atomic plane in the matrix. Scale bar, 2 nm.

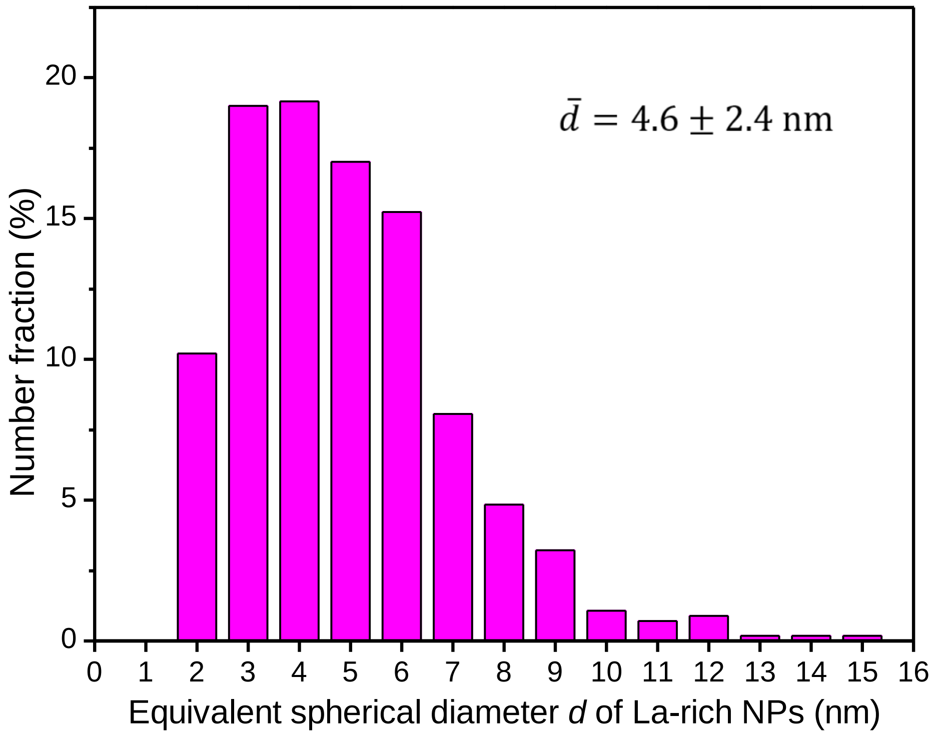

Supplementary Figure 7 | Size distribution of La-riched nanoprecipitates in NC-SS. Size of the La-riched nanoprecipitates is between 2 and 15 nm. The average size of nanoprecipitates is 4.6 ± 2.4 nm. Error for the average size is obtained by the 1st standard deviation of all measurements about nanoprecipitates' size.

Supplementary Tables

Supplementary Table 1 | Chemical composition (wt%) of as-received 304-L CG-SS powder and consolidated NC-SS bulk containing 1at% La.

	Fe	C	Cr	Cu	Mn	La	N	Ni	O	P	Si	W
SS powder	Bal.	0.015	18.59	-	0.19	-	-	10.76	0.31	-	0.84	-
NC-SS	Bal.	0.011	16.69	0.36	0.49	1.66	0.112	13.93	0.30	0.002	0.94	0.53

Supplementary Table 2 | Composition of La,O,Si-rich nanoprecipitates (NPs) and clusters (at%).

	Fe	Cr	Ni	Si	La	W	O
Large NPs (> 8 nm) on GBs	41.41±0.25	13.53±0.14	9.76±0.12	2.58±0.06	17.89±0.17	0.06±0.01	14.88±0.15
Fine NPs (2-8 nm) on GBs	55.18±0.33	18.45±0.19	12.08±0.15	3.18±0.08	6.00±0.11	0.06±0.01	4.82±0.10
NPs (1.5-2.5 nm) in grain interiors	59.45±0.50	15.75±0.26	11.81±0.21	3.08±0.09	5.48±0.13	0.06±0.02	4.00±0.10
Clusters in grain interiors	26.38±0.63	7.47±0.34	4.78±0.27	0.94±0.12	49.58±0.86	-	31.61±0.69

Supplementary Table 3│ Thermodynamic and kinetic properties used in the simulations.

<table>
<tr><td></td><td>E^{m}_{0V}</td><td>E^{m}_{0i}</td><td>U_{0V}</td><td>U_{0i}</td><td>E^{f}_{0V}</td><td>E^{f}_{0i}</td></tr>
<tr><td>Inside grain</td><td>1.4</td><td>0.85</td><td>0.0</td><td>0.0</td><td>1.5</td><td>1.3</td></tr>
<tr><td></td><td>ΔE^{m}_{V}</td><td>ΔE^{m}_{i}</td><td>ΔU_{V}</td><td>ΔU_{i}</td><td>ΔE^{f}_{V}</td><td>ΔE^{f}_{i}</td></tr>
<tr><td>GB</td><td rowspan="2">-0.15</td><td rowspan="2">-0.15</td><td>-0.5</td><td>-0.3</td><td>-0.5</td><td>-0.3</td></tr>
<tr><td>NP interface</td><td>-0.1
-0.5
-0.8</td><td>-0.1
-0.5
-0.8</td><td>-0.1
-0.5
-0.8</td><td>-0.1
-0.5
-0.8</td></tr>
</table>

Migration energy$E^{m}_{V/i}$, formation energy $E^{f}_{V/i}$, binding energy $E_b^{V/i}(lj)$, and chemical potential energy $U_{V/i}$ are in the unit of eV. $E_b^V(lj) = 0.5$ and $E_b^i(lj) = 0.8$ for interstitial cluster *lj*. $E_b^i(lj) = 0.5$ and $E_b^V(lj) = 0.8$ for vacancy cluster *lj*. Formation energies of vacancy and interstitial on dislocations are 1.8 and 1.0 eV, respectively. The thermodynamic and kinetics properties are for austenitic stainless steel[6].

Supplementary References

1. Sun, C., Garner, F. A., Shao, L., Zhang, X. & Maloy, S. A. Influence of injected interstitials on the void swelling in two structural variants of 304L stainless steel induced by self-ion irradiation at 500 ℃. *Nucl. Instru. Methods Phys. Res. B* **409**, 323-327 (2017).
2. Garner, F. A. & Porter, D. L., *Reassessment of the swelling behavior of AISI 304 stainless steel*, p. 5 (United States, 1982).
3. Sun, C., Zheng, S., Wei, C. C., Wu, Y., Shao, L., Yang, Y., Hartwig, K. T., Maloy, S. A., Zinkle, S., J., Allen, T. R., Wang, H. & Zhang, X. Superior radiation-resistant nanoengineered austenitic 304L stainless steel for applications in extreme radiation environments. *Sci. Rep.* **5**, 7801 (2015).
4. Fu, E. G., Misra, A., Wang, H., Shao, L., Zhang, X. Interface enabled defects reduction in helium ion irradiated Cu/V nanolayers, *J. Nucl. Mater.* **407**, 178-188 (2010).
5. Getto, E., Sun, K., Taller, S., Monterrosa, A. M., Jiao, Z., Was, G. S. Methodology for determining void swelling at very high damage under ion irradiation, *J. Nucl. Mater.* **477**, 273-279 (2016).
6. Brimbal, D., Fournier, L. & Barbu, A. Cluster dynamics modeling of the effectof high dose irradiation and helium on the microstructure of austenitic stainless steels. *J. Nucl. Mater.* **468**, 124-139 (2016).
